# Serum IL-33 level is a predictor of progression-free survival after chemotherapy

**DOI:** 10.18632/oncotarget.16627

**Published:** 2017-03-28

**Authors:** Wenwei Hu, Chen Wu, Xiaodong Li, Zhuojun Zheng, Quanqin Xie, Xu Deng, Jingting Jiang, Changping Wu

**Affiliations:** ^1^ Department of Oncology, The Third Affiliated Hospital of Soochow University, Changzhou, Jiangsu Province, China; ^2^ Department of Tumor Biological Treatment, The Third Affiliated Hospital of Soochow University, Changzhou, Jiangsu Province, China; ^3^ Jiangsu Engineering Research Center for Tumor Immunotherapy, Changzhou, Jiangsu Province, China

**Keywords:** IL-33, gastric cancer, chemotherapy, decline extent, progression-free survival

## Abstract

This study aimed to evaluate the association between of serum IL-33 (sIL-33) level in gastric cancer (GC) patients and progression-free survival (PFS). A total of 62 patients with advanced GC and 32 healthy subjects were enrolled. sIL-33 level was detected in pre-chemotherapy patients, post-chemotherapy patients and healthy subjects, respectively. sIL-33 levels were 131.9 (95% CI 105.9-184.9) pg/mL, 95.1 (95% CI 70.8-140.2) pg/mL and 95.7 (95% CI 73.3-114.3) pg/mL in pre-chemotherapy patients, post-chemotherapy patients and controls, respectively. The sIL-33 level in pre-chemotherapy patients was significantly higher than that in both post-chemotherapy patients and controls (*P* < 0.001 and *P* < 0.001, respectively). There was no statistically significant difference between the sIL-33 levels in post-chemotherapy patients and controls (*P* > 0.05). PFS in patients with the decline extent > 30.1% (median PFS not reached) was statistically significant longer than that (median PFS 7 months, 95% CI 1.569 - 12.431) in patients with the decline extent ≤ 30.1% (*P* = 0.003). The decline extent of sIL-33 level (> 30.1%) was associated with longer PFS (*P* = 0.006). Distant metastasis was associated with the decline extent of sIL-33 level (*P* = 0.034). The decline extent of sIL-33 after chemoresistance could be regarded as a predictor of the PFS of GC patients.

## INTRODUCTION

Gastric cancer (GC) is one of the most common malignancies in China, with approximate 380,000 new cases each year. In China, the incidence of GC accounts for more than 40% of the total cases worldwide [1].

The activation of a number of inflammatory cells including myeloid derived suppressor cells and Th2 cells can induce inflammatory reactions and result in the occurrence of chemoresistance [2–4]. Inflammation also activates signal pathways such as just another kinase (JAK), mitogen-activated protein (MAP) kinase, and signal transducer and activator of transcription 3 pathways involved in drug resistance [5, 6].

Interleukin-1 (IL-1) family members are known to alter host response to an inflammatory, infectious, or immunological challenge [7]. IL-33 is a recently identified member of IL-1 family as a potent activator of immune system. A number of studies have demonstrated that IL-33 can promote CD8^+^ T cell function and inhibit cell growth metastasis of tumor cells [8, 9]. IL-33 also induces the production of IL-5 and IL-13 through the activation of intracellular molecules by the nuclear factor-κB and MAP kinase pathway [10]. Ye *et al*. found that IL-33-induced JNK pathway activation was involved in chemoresistance and promoted tumor cell invasion in GC [11]. In clinical practice, the evidence for chemoresistance is disease progression which usually requires changes of chemotherapy regimens.

Besides, sIL-33 level of GC patients has been reported higher than that of healthy subjects, which could be used as a negative predictor of prognosis [12–15]. And there have been reports that high expression of IL-33 is a negative factor for survival of GC patients [12, 16], hepatocellular carcinoma [16] and breast cancer [17].

The purpose of this study was to investigate the expression of serum sIL-33 in patients with GC undergoing chemotherapy and the association with chemoresistance and patients’ prognosis.

## RESULTS

### Enrollment

Excluded those whose follow-up data were not complete, finally a total of 62 GC patients were enrolled from July 2012 and July 2015 in our hospital. Table 1 shows the baseline characteristics of these patients. The median PFS was 13.50 months. Among the patients, there were 51 male and 11 female patients, with a median age of 61 (ranged from 31 to 75) years old. Meanwhile, 32 serum samples from healthy subjects including 25 males and 7 females with a median age of 62 (ranged from 49 to 74) years old were also collected.

**Table 1:** Summary of characteristics for patient with gastric cancer (n=62)

Characteristic	Patient No. (n=62)
Age	
≥ 60 yrs	36 (58.1%)
< 60 yrs	26 (41.9%)
Sex	
Male	51 (82.3%)
Female	11 (17.7%)
Tumor Diameter	
≥ 5 cm	17 (27.4%)
< 5 cm	33 (53.2%)
Unknown	12 (19.4%)
Tumor Location	
Preventriculus	28 (45.2%)
Gastric Corpus	17 (27.4%)
Gastric antrum	15 (24.2%)
Unknown	2 (3.2)
Lymphatic metastasis	
Yes	42 (67.7%)
No	11 (17.7%)
Unknown	9 (14.6%)
Vascular metastasis	
Yes	13 (21.0%)
No	27 (43.5%)
Unknown	22 (35.5%)
AJCC TNM staging	
Stage (I/II)	14 (22.5%)
Stage (III/IV)	43 (69.4%)
Unknown	5 (8.1%)
Distant metastasis	
Yes	20 (32.3%)
No	42 (67.7%)
Chemotherapy	
mFOLFOX6	40 (64.5%)
FOLFIRI	1 (1.6%)
DOF/DCF	20 (32.3%)
None	1 (1.6%)
Pathological grade	
Moderately differentiated; Grade II	43 (69.4%)
Poorly differentiated; Grade III	13 (21.0%)
Unknown	6 (9.6%)
Disease status	
Progression	34 (54.8%)
Stable	28 (45.2%)

### sIL-33 levels

**Figure 1:**
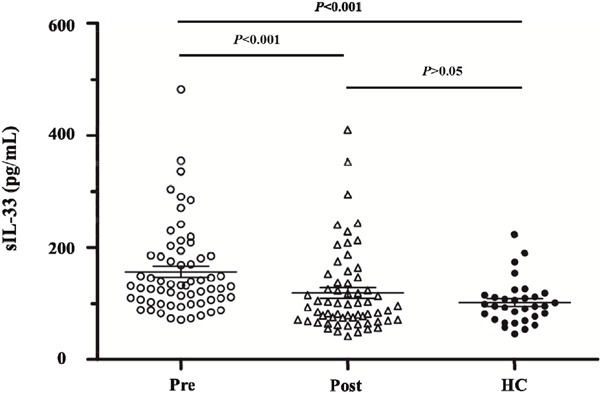
sIL-33 levels are 131.9 (95% CI 105.9-184.9) pg/mL, 95.1 (95% CI 70.8-140.2) pg/mL and 95.7 (95%CI 73.3-114.3) in pre-chemotherapy patients, post-chemotherapy patients and controls, respectively sIL-33 level in pre-chemotherapy patients is significantly higher than that in both post-chemotherapy patients and controls (*P* < 0.001 and *P* < 0.001, respectively). There is no statistically significant difference between the sIL-33 levels in post-chemotherapy patients and controls (*P* > 0.05). Abbreviations: Pre, pre-chemotherapy GC group. Post, post-chemotherapy GC group. HC, healthy control.

As is shown in Figure 1, sIL-33 levels were 131.9 (95% CI 105.9 - 184.9) pg/mL, 95.1 (95% CI 70.8 - 140.2) pg/mL and 95.7 (95% CI 73.3 - 114.3) in pre-chemotherapy patients, post-chemotherapy patients and controls, respectively. The sIL-33 level in pre-chemotherapy patients was significantly higher than that in both post-chemotherapy patients and controls (*P* < 0.001 and *P* < 0.001, respectively). There was no statistically significant difference between the sIL-33 levels in the post-chemotherapy patients and the controls (*P* > 0.05).

### Association between the decline of sIL-33 level and PFS

**Figure 2:**
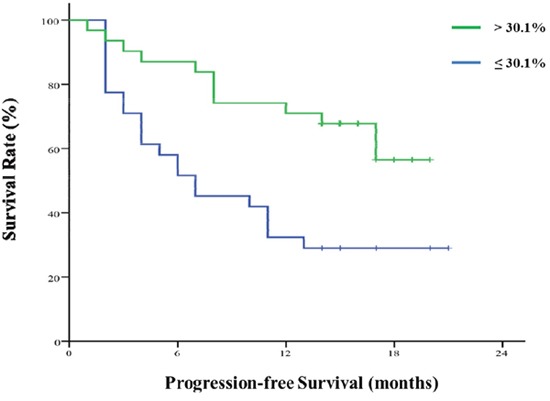
Decline extent of sIL-33 level after chemotherapy and progression-free survival There is a significant difference between the PFS (median PFS not reached) in patients with decline extent > 30.1% and that in patients with decline extent ≤ 30.1% (median PFS 7.0 months). 95% CI 1.569 - 12.431, *P* = 0.003.

Among the 62 patients, sIL-33 level declined in 48 (77.42%). Data were turned into binary variables based on the median decline extent of 30.1%. We investigated the association between decline extent of sIL-33 level and PFS. There was a significant difference between the PFS (median PFS not reached) in patients with decline extent more than 30.1% and that in patients with decline extent no more than 30.1% (median PFS 7.0 months, 95% CI 1.569 - 12.431, *P* = 0.003). See Figure 2.

### Univariate analysis of clinicopathological features influencing PFS

In Table 2, the decline extent of sIL-33 level (> 30.1%) was associated with better PFS (*P* = 0.006). Other clinicopathological features including stage, age, chemotherapy schedule, tumor size, lymph node metastasis, pathological grade, tumor location, distant metastasis showed no associations with PFS (*P* > 0.05).

**Table 2:** Decline extent of sIL-33 after chemotherapy influencing progression-free survival

Clinicopathological feature	Progression		
	HR	95%CI	*P*
Stage (III/IV)	1.013	0.432 – 2.371	0.977
Age (> 60yr)	0.852	0.430 – 1.689	0.646
Chemotherapy (DOF/DCF)	0.693	0.322 – 1.492	0.348
Tumor size (> 5cm)	1.666	0.755 –3.677	0.206
Lymph node metastasis (yes)	0.819	0.329 – 2.042	0.669
Pathological grade (III)	0.549	0.210 – 1.435	0.221
Tumor location			
Gastric corpus	0.690	0.274 – 1.742	0.433
Preventriculus	0.614	0.265 – 1.423	0.256
Decline extent of sIL-33 level (> 30.1%)	0.369	0.181 – 0.752	**0.006**
Distant metastasis	0.806	0.385 – 1.686	0.567

### Regression analysis of clinicopathological features influencing the decline extent of sIL-33 level

In Table 3, distant metastasis was associated with the decline extent of sIL-33 level (*P* = 0.034). Other clinicopathological features including stage, age, chemotherapy schedule, tumor size, lymph node metastasis, pathological grade, tumor location showed no significant differences with the proportion of sIL-33 decline (*P* > 0.05).

**Table 3:** Regression analysis of factors influencing the proportion of sIL-33 decline

Clinicopathological feature	OR	95%CI	*P*
Stage (III/IV)	2.274	0.653– 7.920	0.197
Age (> 60 yr)	1.705	0.616– 4.720	0.305
Chemotherapy (DOF/DCF)	1.833	0.616– 5.453	0.276
Tumor size (> 5cm)	0.944	0.293– 3.048	0.924
Lymph node metastasis (yes)	0.917	0.242– 3.474	0.898
Pathological grade (III)	2.021	0.568– 7.190	0.277
Tumor location			
Gastric corpus	0.467	0.113– 1.920	0.291
Preventriculus	0.769	0.216– 2.745	0.686
Vascular metastasis (yes)	2.423	0.598– 9.816	0.215
Distant metastasis (yes)	3.431	1.100– 10.704	**0.034**

## DISCUSSION

Up to now, there has been no evidence breakthrough for the treatment for advanced GC. The standard regimen of chemotherapy is still under debate [18].

IL-33 is thought to be a dangerous signal of malignant tumor [19], but the role of IL-33 in tumors is complicated that some researchers even hold the opposite view. Gao *et al*. observed that expression of IL-33 inhibited tumor growth and modified the tumor microenvironment through CD8^+^ T and NK cells [9]. Saranchova *et al*. considered that the loss of expression of IL-33 during the metastatic transition actuated immune escape of tumor cells [20]. Tumor development results in downregulation of IL-33 in epithelial cells but upregulation of IL-33 in the tumor stroma and serum, so IL-33 levels in tissue or serum are different [21].

IL-33 in tumor tissues is down-regulated after transient high expression via innate immune regulation. In a few tumor tissues of gastric cancer, lung cancer and colorectal cancer, we have found that IL-33 is of low or negative expression, while of high expression in serum. Serum samples are easier for repeated collection for dynamic observation. Moreover, the window period of IL-33 expression in serum is longer and more stable than that in tumor tissue. So we focused on IL-33 in the study.

We found that the level of sIL-33 in the pre-chemotherapy GC patients was significantly higher than those in post-chemotherapy GC patients and healthy control group. The conclusion was similar to the studies of sIL-33 in lung cancer [22] and endometrial cancer [23]. There was no significant difference between the expression levels of the post-chemotherapy GC patients and the healthy subjects, so sIL-33 is a sensitive indicator of the impact of chemotherapy on the patients. Furthermore, we found the decline extent of sIL-33 after chemotherapy was associated with PFS. If the decline extent of sIL-33 after chemotherapy was more than 30.1%, it predicted a longer PFS. From the univariate analysis of clinicopathological features, the results suggested that the predictive value of the decline extent of sIL-33 level after chemotherapy did not depend on other clinicopathological features. From regression analysis of factors influencing the decline extent of sIL-33, we only found distant metastasis was related to the decline of sIL-33 level. This suggested that sIL-33 level can reflect the effect of the influence of tumor burden on the immune system.

IL-33 is a kind of proinflammatory factor and is inducibly expressed in some stromal cells and infiltrating inflammatory cells. Serum IL-33 (sIL-33) can be detected in healthy subjects and patients with benign or malignant diseases. It has been found that sIL-33 and its receptor soluble ST2 is associated with advanced and metastatic disease in gastric cancer patients and significantly correlates with the duration of the disease. Some gastric cancer patients develop multidrug resistance to chemotherapy. The tumor burdens in these patients do not decrease after chemotherapy and furthermore the immune inhibition even aggravates, which might explain why sIL-33 level increased in some patients in our study.

In our study, the cutoff value was 22.0318pg/ml, based on the sensitivity 0.786 and specificity 55.9. The statistics using the cutoff value also supported the conclusions in the manuscript. In clinical trials, the cutoff value is determined by receiver operating characteristic curves and is the point with the maximum sum of sensitivity and specificity. Due to the small sample size of the current study, there might be large bias of cutoff value, resulting in the failure to represent the whole samples. Moreover, it would lead to a large difference between the 2 group sizes. Thus, the median was used.

sIL-33 is considered to be produced directly by tumor cells or via the immune reactions of lymphocytes to tumor cells. The tumor burden of patients who are sensitive to chemotherapy decreases rapidly and tumor-produced circulating IL-33 level is down-regulated, promoting the Th1/Th2 axis deflecting to Th1, hampering IL-33 inducing Th2 cells, which might be one reason for decreased serum IL-33 level predicting the prognosis.

First-line chemotherapy for gastric cancer is still based on fluorouracil-combined regimens such as FOLFOX, ZELOX and FOLFIRI. The impact of these regimens on survival is not significant and chosen by clinical physicians. Considering these actual background, single regimen in this study was not required. Besides, ToGA trial [24] has confirmed the effect of trastuzumab for Her-2 positive gastric cancer patients, improving the PFS and OS. However, trastuzumab is not an affordable agent for every patient with gastric cancer. In our study, cases with trastuzumab administering were excluded to avoid the bias from receiving trastuzumab or not, and also due to the relative small sample size.

In the current study, the cutoff value was 22.0318pg/ml, based on the sensitivity 0.786 and specificity 55.9. The statistics using the cutoff value also supported the conclusions in the manuscript. In clinical trials, the cutoff value is determined by receiver operating characteristic curves and is the point with the maximum sum of sensitivity and specificity. Due to the small sample size of the current study, there might be large bias of cutoff value, resulting in the failure to represent the whole samples. Moreover, it would lead to a large difference between the 2 group sizes. Thus, the median was used.

In large-scale studies, the factors such as stage, metastasis are found to be related to disease progression [25]. While in the current study the relationship was not found, possibly due to the limited sample size.

Generally, our finding not only confirmed similar results in other tumors [19, 23, 26], but also suggested that the decline extent of sIL-33 after chemotherapy could be an indicator for PFS. Moreover, the detailed mechanisms of sIL-33 level in GC need comprehensive progressive research.

## MATERIALS AND METHODS

### Study design

This was a single-center cohort study. Inclusion criteria included 1) histologically confirmed diagnosis of unresectable advanced GC or metastasis or relapse after operation for GC; 2) a chemotherapy regimen based on 5-fluorouracil (5-Fu)-derived drugs; 3) age of 18 years old or elder; 4) Eastern Cooperative Oncology Group (ECOG) performance status of 0 or 1; and 5) adequate organ function as established with laboratory tests within 7 days of treatment initiation (tests included absolute counts of neutrophil, platelets, hemoglobin, and creatinine, total bilirubin, aspartate aminotransferase, as well as alanine aminotransferase. Exclusion criteria included 1) intolerance to chemotherapy or not receiving chemotherapy due to other reasons; 2) receiving trastuzumab or other targeted drugs; and 3) refusing to receive the detections of HER-2 protein and gene. Data of patients’ clinical features including gender, age, tumor diameter, degree of differentiation, lymph node metastasis, TNM stage, tumor location and histological type were collected. Besides, hospital-based healthy subjects comparable to the cases in demographics were recruited as the control group.

The primary outcome measure was progression-free survival (PFS) time defined as the time from the date of chemotherapy to the date of disease progression inside or outside the stomach or the date of the latest follow-up.

This study complied with the principles of the Declaration of Helsinki and Good Clinical Practice guidelines. All patients provided written informed consents before the enrollment.

### Treatment and sample collection

Enrolled patients received chemotherapy regimens including mFOLFOX6 (oxaliplatin + calcium folinate + 5-Fu), FOLFIRI (irinotecan + calcium folinate + 5-Fu) or DOF/DCF (docetaxel + oxaliplatin + calcium folinate + 5-Fu/docetaxel + cisplatin + 5-Fu). The choice of the chemotherapy regimens was decided by the patient's attending physicians according to the patients’ conditions. After the initial chemotherapy, the CT or MRI scans were performed every two months to evaluate the response to chemotherapy. During the follow-up, if the patient developed suspicious symptoms of disease progression, such as an uptrend of tumor markers like CEA and CA199, unexplained maransis, and newly emerging abdominal discomfort, the researchers would then schedule timely examinations to confirm disease progression. Once the disease progression was confirmed, the follow-up was continued but data not assessed in this study.

Serum samples were collected from patients on day 0 (24 hours before the first cycle of chemotherapy), and 72 hours after the end of the first cycle. Serum samples of the healthy control subjects were collected on the day of physical examinations.

### Detection of sIL-33 level

The detection of sIL-33 was performed by enzyme linked immunosorbent assay method according to the operation manual. The microplate reader (Multiskan GO, Thermo Fisher Scientific Company, Massachusetts, U. S. A.) was used for the measurement of absorbance of all samples. The standard curve was drawn by the standard concentration as the abscissa, and absorbance as the ordinate. Then the sIL-33 concentrations were obtainable from the standard curve.

### Statistical analysis

SPSS Statistics 22.0 (IBM Company, Chicago, U. S. A.) was used for data analysis. Pearson's chi-square test was utilized to compare the constituent ratio in order to analyze the association of sIL-33 level with the clinical features. PFS of various clinical features was compared using Kaplan–Meier and log-rank tests. The Cox proportional hazards model was used to estimate hazard risk (HR) with 95% confidence intervals (CI) of the linking strength between different clinical features and the decline of sIL-33 level before and after chemotherapy. *P* values less than 0.05 were considered statistically significant. The sIL-33 level was described as median (25^th^ and 75^th^ percentile) as it was not in accordance with Gaussian distribution, and thus was compared by one-way ANOVA and post-hoc-analysis. All statistical tests were two-sided. GraphPad Prism 6.02 (CA 92037, USA) was utilized for creating statistical graphs.
